# Management of Chronic Symptoms of Lyme Disease With Intravenous Ceftriaxone

**DOI:** 10.7759/cureus.16354

**Published:** 2021-07-13

**Authors:** Muhammad Naeem, Derek Enos, Shazia Shah, Nidhi Patel, Tara Fisher

**Affiliations:** 1 Internal Medicine, St. Francis Medical Center, Trenton, USA; 2 Medicine, Drexel University College of Medicine, Philadelphia, USA; 3 Medicine, Seton Hall University Hackensack Meridian School of Medicine, Philadelphia, USA

**Keywords:** lyme disease, oral doxycycline, iv ceftriaxone, management of chronic symptoms of lyme disease, lyme's disease, lyme disease and other tick borne pathogens

## Abstract

Lyme disease is a vector-borne illness of North America and Europe transmitted by *Borrelia burgdorferi*, over 30,000 cases are reported in the United States yearly. Patients typically present having early localized disease with fevers, headaches, myalgias, and a single erythema migrans. Usually, oral doxycycline is administered with a good disease prognosis but we report the case of a 58-year-old male who presented with Lyme disease diagnosed by immunoassay; he was treated with doxycycline but was refractory and saw an improvement in his symptoms with IV ceftriaxone.

## Introduction

Lyme disease is a vector-borne illness of North America and Europe transmitted by Borrelia burgdorferi, a spirochete organism carried by Ixodes species of deer ticks [1]. Although the risk of developing Lyme disease after being bitten by an infected tick ranges from 1% to 6% [2], over 30,000 cases are reported in the United States yearly [1]. Patients typically present in early localized disease with fevers, headaches, myalgias, and a single erythema migrans. Of those who presented, treatment including amoxicillin, azithromycin, cefuroxime, and, most commonly, doxycycline is administered with a good disease prognosis. However, erythema migrans may be subtle and go unnoticed, having the disease proceed to early disseminated Lyme disease occurring weeks to months later, causing complications of carditis, neurologic infections, and ocular and articular manifestations. The prognosis of symptom resolution continues to prove to be good, especially when treated with doxycycline or ceftriaxone. Late disseminated Lyme disease, over three months after not receiving treatment, evolves into persistent arthritis and neuroborreliosis, which includes meningeal symptoms or encephalopathy, facial paralysis, and Bell’s palsy, radiculopathy, and cognitive declines [2].

The early localized disease is distinguished by the red ring-like expanding rash of erythema migrans at the site of a recent tick bite. Most patients only experience the symptoms of early, localized disease. About 20% of patients develop the early disseminated disease, with the most common symptoms being multiple erythema migrans lesions [3]. Other symptoms of the disseminated stage are flu-like symptoms, lymphadenopathy, arthralgia, myalgia, palsies of the cranial nerves (especially CN-VII), ophthalmic conditions, and lymphocytic meningitis. Additionally, cardiac manifestations such as conduction abnormalities, myocarditis, or pericarditis may occur. The most common manifestation of the late disease is arthritis that is usually pauciarticular and affects large joints, especially the knees [3]. If suspected, a two-test system is implemented for the diagnosis of Lyme disease, including an initial enzyme immunoassay or immunofluorescence assay followed by a Western immunoblot assay if initial results were positive or equivocal [4-5]. Typically, an immunoglobulin M (IgM) blot would produce two out of three Lyme-specific bands for positivity while an immunoglobulin G (IgG) blot would produce five out of 10 Lyme-specific bands [4].

The mainstay of treatment of early Lyme disease, both localized and disseminated, is oral doxycycline. For individuals in whom doxycycline is contraindicated, viable alternatives include amoxicillin and cefuroxime. Complete resolution of all symptoms with any of these three drugs approaches 90% [6]. Studies have shown that extended IV antibiotic therapy is associated with low morbidity and no mortality in patients referred for treatment of neurologic Lyme disease [7]. We report the case of a 58-year-old male who presented with Lyme disease diagnosed by immunoassay who was treated with doxycycline but was refractory and saw an improvement in his symptoms with IV ceftriaxone.

## Case presentation

We present a 58-year-old Middle Eastern man with a past medical history of Lyme disease treated for three months with oral doxycycline 100 mg BID in March 2020 diagnosed by strongly positive serology by enzyme linked immunosorbent assays (EIA) (and both IgG and IgM Western blotting from a reputed lab but did not respond to treatment. Further past medical history includes traumatic brain injury with subsequent subdural hematomas and progressive leg weakness due to falls in 2016, cervical and lumbar discogenic disease, compression fractures of T8 and T12, hypertension, and depression present with a two-week history of worsening weakness and fatigue associated with diffuse, aching joint pain of his knees, hips, shoulders, elbows, heels, and toes, radicular pain radiating through bilateral lower extremities, numbness and paresthesias of his left second, third, and fourth toes, decreased strength, inability to ambulate, sporadic muscle spasms, decrease in the ability to balance, and clumsiness with movement. He additionally reports headaches, dizziness, short-term and long-term memory decline, inattentiveness, worsening depression without suicidal ideation, and speech difficulties. The patient denies any paralysis, nervousness, anxiety, hallucinations, or irritability. The patient noticed these progressive symptoms in 2016 after multiple falls and has noticed a significant decline in the last two weeks. He additionally reported mild improvement after his doxycycline therapy that reverted to his current state. He was previously a truck driver in the Northeastern United States. His only allergy was iodinated contrast. He denied any smoking, alcohol, or tobacco use, and his family history was noncontributory.

On examination, the patient was frail appearing and not in acute distress. He experienced neck stiffness with decreased flexion and extension along with pain during right and left rotation. He experienced mild cervical tenderness radiating to both shoulders. Mild crackles were auscultated in right lung fields with the left lung fields clear to auscultation. A regular heart rate was auscultated with no murmurs, rubs, and gallops. The patient was alert and oriented to person, place and time, and cranial nerves II through XII were intact. He did not display Bell's palsy. His extremities all displayed 4-/5 strength. His deep tendon reflexes (DTRs) were 0+ on the biceps, triceps, and knees and otherwise 1+. Gait was unable to be assessed due to the patient’s fatigue. His sensations were intact in the extremities. The remainder of the physical exam was unremarkable.

His laboratory diagnostics of hematology with differential, chemistry, and urinalysis on admission were unremarkable except for mildly elevated blood glucose of 101 mg/dL and blood eosinophils of 6.1%. His chest X-ray and CT scan on admission were unremarkable. He, however, did present with outpatient MRI that displayed lumbar discogenic disease at the L4-L5 level with nerve root impingement and central stenosis and laboratory elevated IgM and IgG antibodies to Lyme disease.

The patient was admitted to the inpatient medical service to be evaluated by infectious disease and neurology who decided to perform a lumbar puncture and peripherally inserted central catheter (PICC) line placement to receive IV ceftriaxone 2 g daily therapy for four weeks. His lumbar puncture displayed a cloudy, pinkish-red sample with high red blood cells at 9660, white blood cells (WBC) high at 13 with 100% neutrophils in differential, the glucose of 68, and total protein high at 85. Routine and fungal cultures of the cerebrospinal fluid (CSF) were negative. He was ultimately discharged on the fourth day of stay after receiving two days of ceftriaxone with subjective improvements in arthralgias and fatigue and displaying 5/5 strength in all extremities, +1 DTRs, and ability to ambulate longer distances without assistance.

**Figure 1:**
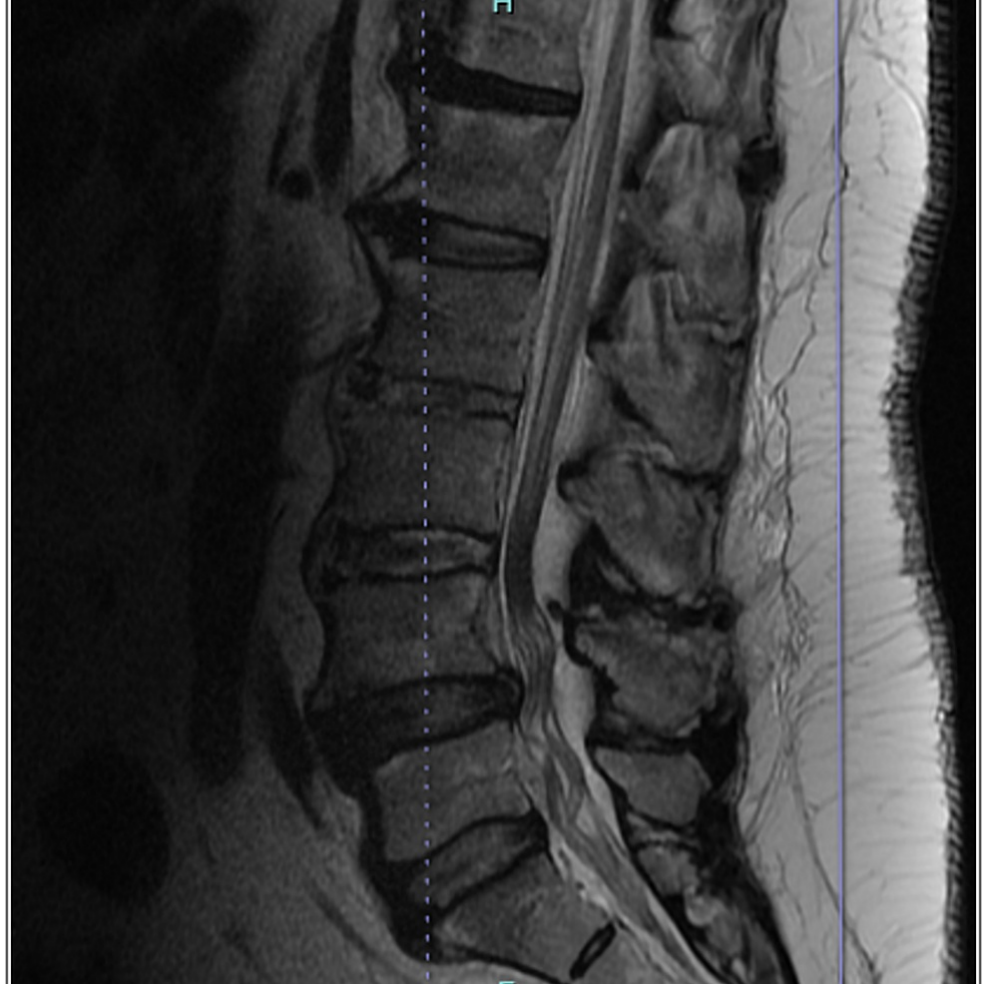
MRI lumbar spine w/wo contrast - shows normal conus medullaris. No unusual contrast enhancement through the cauda equina or proximal nerve roots to suggest neuromyelitis. Multilevel lumbar foraminal stenosis. Clumping of the nerve roots at L3-L4 is a nonspecific finding. There is no contrast enhancement or focal mass. This may be evidence of scarring from focal arachnoiditis. Degenerative disc disease throughout the upper lumbar spine consists primarily of anterior osteophyte formation. No disc herniation.

After discharge, the patient’s HIV1 and 2 antibodies with P24 antigen, rapid plasma reagin (RPR), cryptococcus, angiotensin converting enzyme (ACE) level, complement C3, C4, and CH50, cardiolipin IgA, IgG, IgM antibodies, phospholipid antibodies, anti-dsDNA, and Quantiferon Gold all returned within normal limits. His Lyme antibody was high, greater than 12, and his Lyme Western Blot displayed reactive 18, 28, 30, 39, 41, 58 KG IgG bands with non-reactive IgM bands. His CSF analysis for Borrelia species was negative. Additionally, CSF studies of venereal disease research laboratory (VDRL), IgG, and albumin were all within normal limits. He did display oligoclonal bands of five identical gamma restriction bands indicative of systemic rather than intracerebral synthesis of gamma globulins. His MRI with and without contrast displayed degenerative disc disease of the upper lumbar spine primarily of osteophyte formation without disc herniation or canal stenosis (Figure 1).

## Discussion

Lyme disease or Lyme borreliosis is the most commonly transmitted tick-borne infection in the United States and can be divided into three stages: early localized, early disseminated, and late disseminated. The diagnosis is not easy as many patients are unable to recall a tick bite. However, Lyme disease should be suspected in individuals manifesting symptoms who are traveling to or living in endemic areas and should be started on therapy. The typical features of late disseminated disease and untreated infection include neurological and rheumatological involvement. The key features of this stage of Lyme disease are arthritis, which primarily affects the knee, and neurological and psychiatric symptoms that mimic fibromyalgia [5]. Similarly, radicular pain is common. The patient's case above presented with progressive symptoms of diffuse, aching joint pain of his knees, hips, shoulders, elbows, heels, and toes led us to suspect chronic Lyme as a possible etiology.

There are no standardized diagnostic criteria for Lyme borreliosis. Unfortunately, this has led to both over- and under-diagnosis of Lyme disease. The disease requires the tick, the spirochete, and the host. In other words, the presence of ticks by themselves does not in any fashion lead to the diagnosis of Lyme disease [8]. The condition of most patients with Lyme disease improves after initial antibiotic therapy which consists of a specific treatment depending upon the age and stage of the disease. Although patients older than eight years of age should receive doxycycline for the early, localized phase of infection, patients under the age of eight or pregnant patients should receive amoxicillin and ceftriaxone, respectively [5]. About 10%-20% of patients treated for Lyme disease may have lingering symptoms of profound and debilitating fatigue, asymmetrical musculoskeletal pains, disrupted sleep, and lack of customary mental functions such as verbal memory deficits, and poor auditory attention [9].

When symptoms are continuous or relapsing for at least six months following the completion of antibiotic treatment and severe enough to reduce functional ability in a patient’s life, the likely etiology is a non-infectious, post-treatment Lyme disease syndrome (PTLDS). The pathogenesis of PTLDS is unknown, although elevated levels of IL-23 or CCL19 have been reported [10]. However, the Infectious Disease Society of America (IDSA) defines PTLDS as a documented episode of early or late Lyme disease with post-treatment resolution of objective symptoms but with the subsequent onset of symptoms of fatigue, widespread musculoskeletal pain, and/or complaints of cognitive difficulties [11]. Patients with PTLDS recover from persistent symptoms with time, however, it can take months before the patient feels well. Reinfection with *Borrelia burgdorferi* can often be recognized clinically by the development of a repeat episode of erythema migrans occurring at a different skin site during months when the nymphal stage of the principal tick vectors is plentiful in the environment. Reinfection is more reliably diagnosed in patients with recurrent erythema migrans lesions than in patients with extracutaneous manifestations of the disease [12].

In this patient, the joint pain and neuropathic symptoms returned after discharge although not as severe as pretreatment. The patient's IV ceftriaxone treatment was stopped after three weeks because he developed a blood clot related to his PICC line and was lost to follow-up. The patient's prior physician treated his joint symptoms and did not recognize that the patient had any neurological Lyme disease, he was told to have neurological symptoms due to discogenic disease. The patient did not have any significant discogenic disease although he had multilevel lumbar foraminal stenosis and nerve root clumping. This patient failed to respond to oral doxycycline for neurological Lyme disease. The patient had several physical findings suggestive of peripheral neuropathy (weakness, diminished reflex, lower extremity vibratory sense were essentially absent). An electromyography and nerve conduction velocity (EMG-NCV) was never performed because he was lost to follow-up. His lumbar puncture was traumatic and that explains high WBC, but not the WBC differential for high CSF protein.

The oligoclonal bands are consistent with systemic inflammation. The MRI suggested arachnoiditis due to nerve root clumping. In Europe, neurological Lyme disease is treated with oral therapy but their strains are different compared to US strains. There are no comparative studies between IV and oral therapy for US strains. This article’s contribution is a warning about the possible lack of effectiveness of oral doxycycline for neurological infections. The newly released national guidelines from the IDSA discussed the treatment issues in detail but the hypothesis about doxycycline efficacy for US treatment is based on in vitro susceptibility testing. The in vitro data does not have the same quality evidence as clinical studies.

## Conclusions

Fewer than 10% of cases of central nervous system (CNS) Lyme disease will demonstrate predominantly neutrophils in CSF, because this finding is atypical one must consider other diagnoses, such as CNS vasculitis and other connective tissue diseases. An auto-immune evaluation was performed as an outpatient and was not revealing. An outpatient MRI scan of the brain with and without contrast performed within the past six weeks was normal. In the end, we may just conclude that the CSF finding was a reflection of Lyme disease, IV ceftriaxone is entirely appropriate and warranted due to better outcomes.
